# Humoral immune responses in hospitalized COVID-19 patients

**DOI:** 10.1016/j.sjbs.2021.04.032

**Published:** 2021-04-20

**Authors:** Waleed H. Mahallawi

**Affiliations:** Medical Laboratory Technology Department, College of Applied Medical Sciences, Taibah University, Madinah, Saudi ArabiaMedical Laboratory Technology Department, College of Applied Medical Sciences, Taibah University, Madinah 41541, Saudi Arabia

**Keywords:** SARS-CoV-2, Severe, Non-severe, Humoral immunity, Hospitalized

## Abstract

**Background:**

The emerging coronavirus 2019 (COVID-19) disease, caused by infection with severe acute respiratory syndrome coronavirus 2 (SARS-CoV-2), is a worldwide public health crisis. Antibody analysis is an important procedure for the diagnosis of COVID-19 patients. We investigated the IgG, IgM, and IgA responses against the SARS-CoV-2 spike (S) protein among hospitalized COVID-19 patients.

**Materials and methods:**

Hospitalized COVID-19 patients (n = 178) in the Al Madinah region, Saudi Arabia, participated in this study. Of the 178 patients, 72 (40%) were categorized as severe, including 50 (69%) males and 22 (31%) females. The remaining106 (60%) patients were categorized as non-severe, including 85 (80%) males and 21 (20%) females. Qualitative reverse transcription-polymerase chain reaction (RT-PCR) to detect the presence of SARS-CoV-2 RNA was used to confirm the diagnosis of each patient. The specific anti-SARS-CoV-2 S protein IgG, IgM, and IgA antibodies in patients’ sera were measured using enzyme-linked immunosorbent assay (ELISA) and compared between case presentations.

**Results:**

The current study showed that all severe hospitalized patients presented significantly (p < 0.0001) increased anti-S IgG and IgM antibody accumulation compared with non-severe patients. Additionally, the results also showed that 50% of severe males were positive to anti-S IgG, IgM, and IgA antibodies, whereas only 40% positivity for all three-antibody isotypes was observed in severe females. The study also showed that 86% of males and 81% of females categorized as severe were positive for both IgG and IgM antibodies but negative for the IgA antibody against the S protein.

**Conclusion:**

The humoral immune response against SARS-CoV-2 proteins commonly results in the production of antibodies against viral proteins. Specific anti-SARS-CoV-2 S protein IgG class antibodies were detected at significantly higher levels than IgM class antibodies, and both IgG and IgM antibodies were detected at significantly higher levels than the IgA antibody among all patients. The variations of the humoral immune responses among hospitalized patients reflect the association between disease presentations and immunity against the virus. Collectively, these findings afford new insights into the different antibody isotypes in responses to COVID-19 hospitalized patients with dissimilar disease severity.

## 1 Introduction

In late 2019, in China’s Hubei province, cases of pneumonia with an unknown cause were reported (Chen et al., 2020). Later, the global incidence of severe acute respiratory syndrome (SARS) was confirmed to be associated with a new coronavirus, termed SARS coronavirus 2 (SARS-CoV-2), which causes a disease now known as coronavirus disease 2019 (COVID-19) (Cordes & Heim, 2020). COVID-19 causes a wide variety of mild-to-severe symptoms, with hospitalization in intensive care units (ICUs) necessary for the most severe cases (Li et al., 2020, Wu et al., 2020). Antibodies against SARS-CoV-2 proteins are generated by the humoral immune response. Virus-specific IgM antibodies are produced first, followed by the more specific IgG antibodies (Liu et al., 2021, Long et al., 2020a). The IgA response against SARS-CoV-2 has been reported to be both rapid and persistent (Padoan et al., 2020) and has been associated with mucosal responses, including both respiratory and gastrointestinal responses (Isho et al., 2020).

Reports have indicated that 80% of COVID-19 infections result in mild or asymptomatic disease manifestations, whereas approximately 15% are severe or require oxygen supplementation (Borobia et al., 2020). Nearly 5%–10% are categorized as critical, associated with acute respiratory distress syndrome (ARDS) and necessitating mechanical ventilation in an ICU (Yang et al., 2020). One distinctive characteristic of SARS-CoV-2 is that, globally, children and young people tend to present with a milder disease manifestation with a significantly reduced frequency of ARDS, which is a hallmark of COVID-19 morbidity (Otto et al., 2020).

The fraction of patients with severe COVID-19 that require ICU treatment has ranged from 4% to 32% (Aziz et al., 2020), and worries that ICU capacities may be overwhelmed have resulted in serious deliberations regarding the implementation of lockdowns and social distancing guidelines (Eubank et al., 2020).

Antibodies have the critical capacity to neutralize viruses and protect the host against viral infection. The spike (S) protein of SARS-CoV-2 interacts with the angiotensin-converting enzyme 2 (ACE2) on host cells to induce viral entry. Thus, targeting this receptor is viewed as a potential therapeutic solution for preventing viral entry into host cells and inhibiting infection (Chan et al., 2020).

The kinetics of immune response relative to both clinical and virological features in patients with varying clinical COVID-19 presentations, including those who require hospitalization, has not yet been fully studied. As the global COVID-19 pandemic continues, comprehensive investigations into the immune response of COVID-19 patients become an increasingly pressing need. The precise description of specific antibody response kinetics is fundamental to our understanding of the mechanisms that determine disease severity and outcomes (Rodriguez-Morales et al., 2020).

SARS-CoV-2 can infect all age groups, presenting a range of disease manifestations, including asymptomatic, mild, moderate, and severe symptoms, with potentially fatal consequences (Jin et al., 2020, Mahallawi, 2021). Although the causes of these variable disease presentations may be associated with numerous aspects, including age, sex, pre-existing comorbidities, or host genetics, the host immune response is likely to be a contributor that influences infection outcomes (Cai, 2020, Castagnoli et al., 2020, Williams et al., 2021).

The present study aimed to investigate the humoral immune response among hospitalized COVID-19 patients, compared across different clinical presentations. Enzyme-linked immunosorbent assay (ELISA) was used for the simultaneous measurement of anti-S-specific IgG, IgM, and IgA antibody levels in the sera of COVID-19 patients. The relationship between disease severity, patients' presentations and the immune response among hospitalized patients would be of interest for more investigation.

## 2 Materials and methods

### 2.1 Patient samples

Hospitalized COVID-19 patients (n = 178) were enrolled in this cross-sectional study. All patients a signed written consent form before the study was conducted. Samples were collected between April 14, 2020, and May 25, 2020. All the samples were collected 7 to 10 days following hospital admission. This study was approved by the research ethics committee of the General Directorate of Health Affairs in Al Madinah (IRB number: 496).

## 3 RT-PCR test for SARS-CoV-2

Nasopharyngeal (NP) swabs were collected using sterile swabs (BD, USA). The swab was maintained in the NP cavity for a few seconds to allow the absorption of nasal secretions. The swab was then immediately transferred into sterile tubes containing 2–3 ml of viral transport media (VTM) for further processing (Radbel et al., 2020).

## 4 RNA extraction

The extraction of RNA was performed using a Roche Magna Pure LC (RNA Viral Isolation Kit, USA). A 200 µL volume of each sample was loaded onto a MagNA pure LC 96-well plate. Reaction reagents were then added and verified prior to extracting the samples, as described by the manufacturer’s instructions for nucleic acid extractions from specimens. For a detailed protocol, see https://www.moh.gov.sa/Ministry/MediaCenter/Publications/Documents/Coronavirus-Disease-2019-Guidelines-v1.2.pdf

## 5 The one-step RT-PCR real-time amplification

After viral RNA extraction, reverse transcription-polymerase chain reaction (RT-PCR) was performed using an Altona Diagnostics RealStar® SARS-Cov-2 RT-PCR detection kit 1.0, targeting the specific RNA SARS-Cov-2 Envelope gene (E gene) and the SARS-Cov-2 S gene based on the one-step RT-PCR real-time method. All tubes containing one-step RT-PCR mixtures were closed and cautiously moved to a Real-Time LC 480 (Roche, USA). The following program was used: a single cycle at 55 °C for 20 min, a single cycle at 95 °C for 2 min, and 45 cycles at 95 °C for 15 s, 55 °C for 45 s, and 72 °C for 15 s. The one-step RT-PCR assay was performed in a negative pressure cleanroom for extraction to prevent contamination. www.altona-diagnostics.com/en/products/reagents-140/reagents/realstar-real-time-pcr-reagents/realstar-sars-cov-2-rt-pcr-kit-ruo.html.

## 6 Enzyme-linked immunosorbent assay (ELISA)

An ELISA was performed to detect antibodies against the SARS-CoV-2 S protein, following a previously described protocol (Mahallawi, 2020). Briefly, a 96-well ELISA plate (Costar; Corning, Corning, NY, USA) was coated with the SARS-CoV-2 recombinant S protein (Sino Biological, Beijing, China). The SARS-CoV-2 S protein was dissolved in phosphate-buffered saline (PBS; pH 7.2) and 100 µL SARS-CoV-2 S protein, at 2 µg/mL, was added to each well. The plates were then covered with an adhesive seal and stored overnight at 4 °C. The plate contents were discarded, and the plates were washed five times with washing buffer (PBS containing 0.05% Tween-20; Sigma-Aldrich, St. Louis, MO, USA). The plates were then blocked with 150 µL/well blocking buffer (PBS containing 0.05% foetal bovine serum (FBS) that was heat-inactivated at 56 °C for an hour; Sigma-Aldrich) for one hour at room temperature. Serum samples were diluted (1:100) using blocking buffer, and 100 µL diluted sample was added to each well. The plates were incubated for 30 min at room temperature and then washed five times with washing buffer. Alkaline phosphatase-conjugated goat anti-human secondary antibodies (IgG, IgM, and IgA at 1:1,000, 1:2,000, and 1:1,000, respectively, in blocking buffer, Sigma-Aldrich) were then added at 100 µL/well, and the plates were incubated at room temperature for 30 min before being washed five times. All wash steps were performed using an automated microplate washer (Elx50; Bio Tek, Winooski, VT, USA). Finally, 100 µL/well of, p-nitrophenyl phosphate substrate (p-NPP, Sigma-Aldrich), was added. The plates were stored in the dark, away from direct light. After 30 min, 100 µL of stopping solution (1.2 N sodium hydroxide, Reagecon, UK) was added to all wells to stop the reaction. The optical density (OD) at 405 nm was measured using a microplate reader (ELX800; BioTek).

### 6.1 Statistical analysis

GraphPad Prism software version 9 (GraphPad, San Diego) was used to perform statistical analyses. For the measurement of anti-SARS-CoV-2 Spike protein antibodies among all patients, mean was used. Other variables were expressed as the mean ± standard error of the mean (SEM). Spearman’s correlation coefficient analysis was used to measure the correlation between two variables. P-values were determined using an unpaired, two-sided Mann–Whitney *U* test. A P-value of < 0.05 was considered significant.

## 7 Results

### 7.1 Demographic data of the patients

The positive detection of SARS-CoV-2 RNA was confirmed in all patients using qualitative RT-PCR. Patients were divided into two groups according to the disease severity: patients with mild-to-moderate symptoms (non-severe group) and patients with severe symptoms (severe group). Patients were classified based on the ministry of health (MOH) guidelines (https://www.moh.gov.sa/Ministry/MediaCenter/Publications/Documents/MOH-therapeutic-protocol-for-COVID-19.pdf, accessed on 05.02.2021), according to the detailed medical records obtained from the hospital.

The recruited patients were hospitalized COVID-19 patients (n = 178) from the Al Madinah region, Saudi Arabia. Among the 72 severe patients (40%) 50 were male, (69%%, mean age of 38 years) and 22 were female (31%, mean age of 33 years). Among the 106 (60%) non-severe patients, 85 were male (80%, mean age of 42 years) and 21 were female (20%, mean age of 39 years).

## 8 Measurement of anti-SARS-CoV-2 spike protein antibodies

**Fig. 1:**
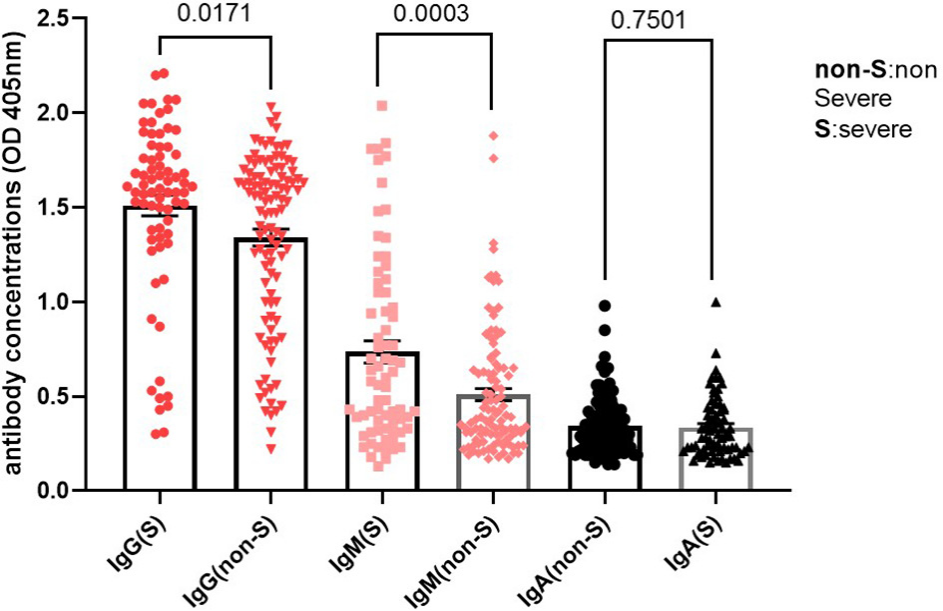
**Anti-S IgG, IgM and IgA antibody concentrations in severe (S) and non-severe (NS) patients.** The anti-S antibody isotype concentrations in serum of severe (n = 72) and non-severe (n = 106) COVID-19 patients were determined by ELISA and expressed in units of optical density (OD at 405 nm). The p values between the different groups are indicated.

To study the humoral immune responses of the hospitalized patients, ELISA was used to measure specific anti-S IgG, IgM, and IgA serum antibody concentrations (in units of optical density). Fig. 1 shows that the mean anti-S IgG antibody concentration of severe patients (mean = 1.51, n = 72) was significantly higher than that of non-severe patients (mean = 1.34, n = 106, p = 0.017). Additionally, the results showed that the mean anti-S IgM antibody concentration of severe patients (mean = 0.74, n = 72) was significantly higher than that of non-severe patients (mean = 0.51, n = 106, p = 0.0003). However no significant difference in the mean anti-S IgA antibody concentrations was observed between the severe (mean = 0.33, n = 72) and non-severe (mean = 0.34, n = 106) patients (p = 0.75).

## 9 Correlation between different anti-S antibody classes in COVID-19 patients

**Fig. 2:**
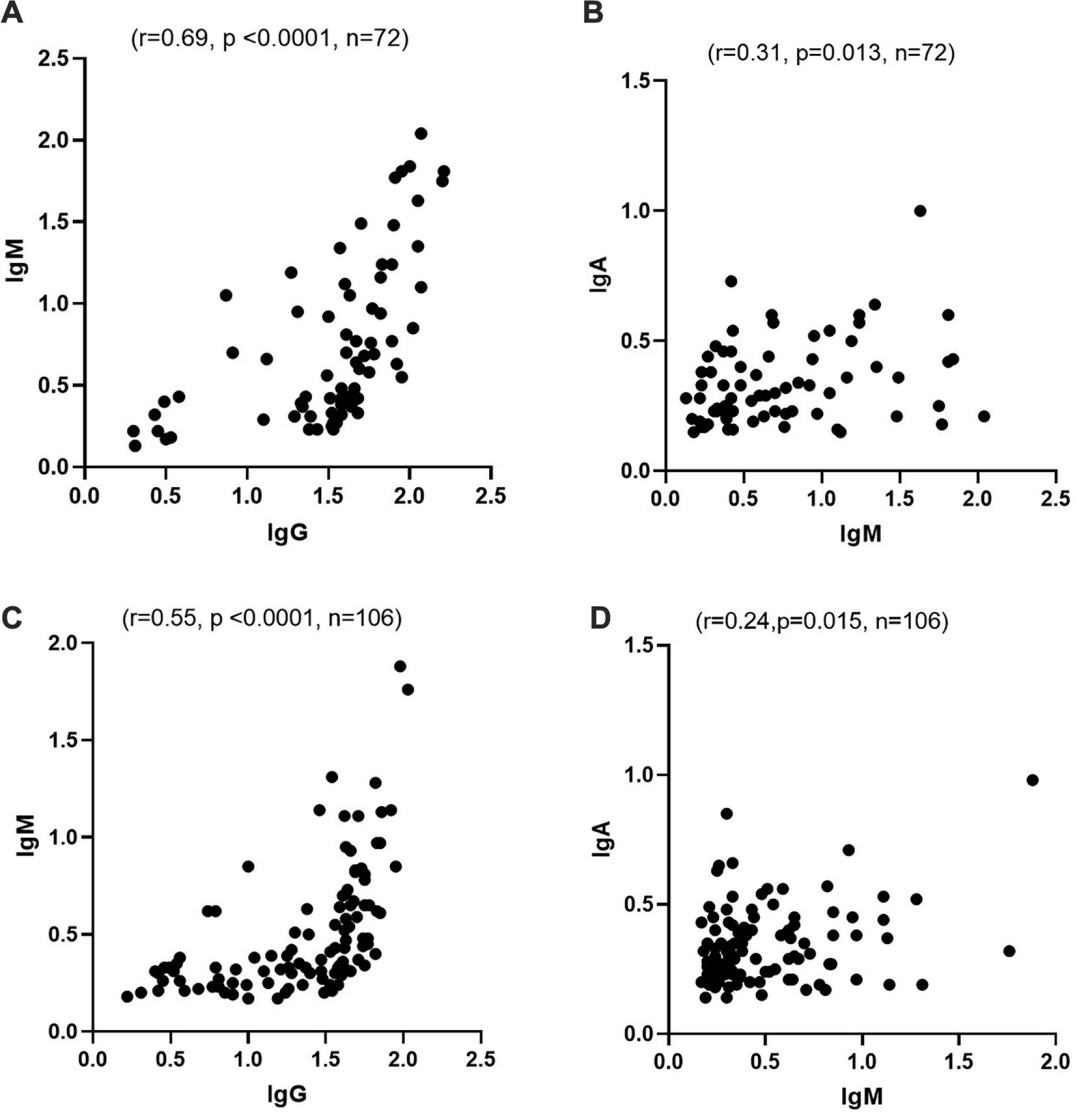
Correlation between anti-S antibody isotypes in severe and non-severe patients. (**a**) A strong positive correlation between anti-S IgG and IgM antibodies in severe patients (Spearman’s r = 0.69, p < 0.0001, n = 72). (**b**) A positive correlation between anti-S IgM and IgA antibodies in severe patients (Spearman’s r = 0.31, p = 0.013, n = 72). (**c**) A strong positive correlation between anti-S IgG and IgM antibodies in non-severe patients (Spearman’s r = 0.55, p < 0.0001, n = 106). (**d**) A positive correlation between anti-S IgM and IgA antibodies in non-severe patients (Spearman’s r = 0.24, p = 0.015, n = 106).

To determine whether any correlation exists among the antibody levels in COVID-19 patients, the results for the three classes of anti-S antibody were compared within each patient category. In severe patients, a strong positive correlation was observed between anti-S IgG and IgM antibodies (Spearman’s r = 0.69; 95% confidence interval 0.54 to 0.79, p < 0.0001, n = 72, Fig. 2(a)). A positive correlation was also observed between anti-S IgM and IgA antibody concentrations in severe patients (Spearman’s r = 0.31, 95% confidence interval 0.059 to 0.5, p = 0.013, n = 72, Fig. 2(b)). In non-severe patients, a strong positive correlation was observed between anti-S IgG and IgM antibody concentrations (Spearman’s r = 0.55, 95% confidence interval 0.40 to 0.67, p < 0.0001, n = 106, Fig. 2(c)). A positive correlation was also observed between anti-S IgM and IgA antibody concentrations in the non-severe group (Spearman’s r = 0.24, 95% confidence interval 0.047 to 0.401, p = 0.015, n = 106, Fig. 2(d)). No correlation was observed between antibody concentrations and patients’ age (data not shown).

## 10 Antibody concentrations and patients’ sex

**Fig. 3:**
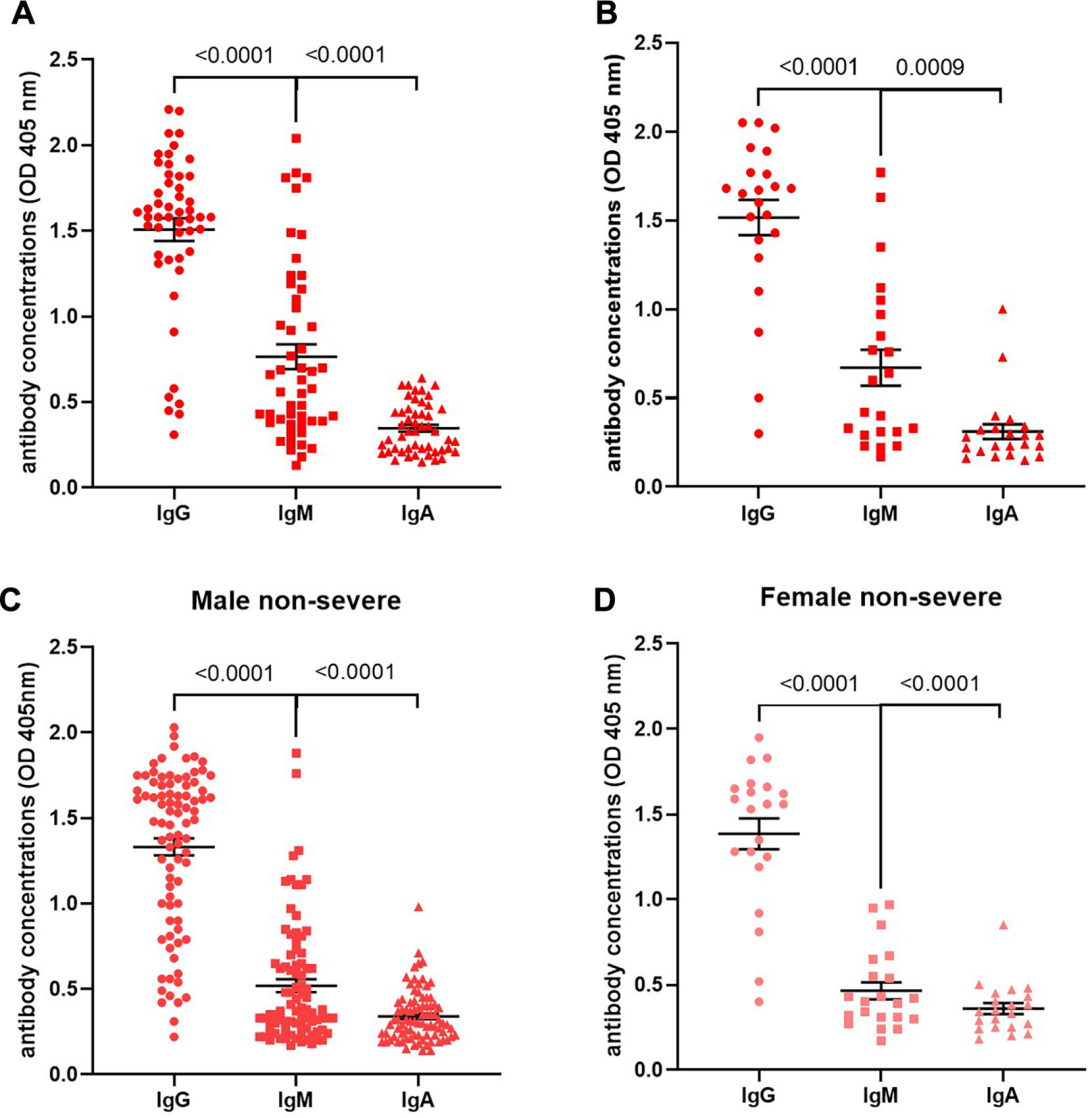
Serum anti-S antibody isotype concentrations in severe and non-severe patients as measured by ELISA. (**a**) Anti-S IgG, IgM and IgA antibody concentrations of severe male patients (n = 50). (**b**) Anti-S IgG, IgM and IgA antibody concentrations of severe female patients (n = 22). (**c**) Anti-S IgG, IgM and IgA antibody concentrations in non-severe male patients (n = 85). (**d**) Anti-S IgG, IgM and IgA antibody concentrations in non-severe female patients (n = 21). The p values between the different groups are indicated.

To investigate the differences between humoral immune response in male and female patients, antibody concentrations were measured by ELISA and compared within each sex. Fig. 3(a) shows that the anti-S IgG (p < 0.0001, n = 50) antibody concentration (in units of OD) of severe male patients was significantly higher than the anti-S IgM antibody concentration, and both were significantly higher (p < 0.0001, n = 50) than the IgA antibody concentration. A similar trend was observed in severe female patients (Fig. 3(b)).

Additionally, Fig. 3(c) shows that the anti-S IgG antibody concentration of non-severe male patients was significantly higher (p < 0.0001, n = 85) than the anti-S IgM antibody concentration, and both were significantly higher (p < 0.0001, n = 85) than the IgA antibody concentration. A similar trend was observed in non-severe female patients (Fig. 3(d)). The anti-S of IgG, IgM, and IgA levels according to the various patients’ categories are summarized in Table 1.

**Table 1:** Antibody concentrations against the viral S protein. Percentages of anti-S IgG, IgM, and IgA isotypes stratified by sex and disease severity.

IgG(-), IgM(-) IgA(-)%	IgG(+), IgM(-) IgA(-)%	IgG(+), IgM(-) IgA(+)%	IgG(+), IgM(+) IgA(-)%	IgG(+), IgM(+) IgA(+)%	Disease Severity	Gender
2	8	4	86	50	**Severe (n = 50)**	**Male**
4.5	9	9	81	40	**Severe (n = 22)**	**Female**
1	16.5	3.5	32	43	**(non-severe) (n = 85)**	**Male**
0	9.5	9.5	28.5	52	**(non-severe) (n = 21)**	**Female**

## 11 Discussion

The humoral immune response against SARS-CoV-2 proteins generally results in the production of antibodies against viral proteins. Cohorts of hospitalized patients diagnosed with COVID-19 have been identified globally; however, to date, limited data from Saudi Arabia have been available (Al Mutair et al., 2020, Shabrawishi et al., 2020). Studying the humoral immune responses in hospitalized patients is an important issue for both clinical and research purposes. Correlating the disease severity with the antibody response remains a point of interest that has not been fully studied or well-elaborated in most clinical settings, especially in Saudi Arabia. Therefore, the results of such studies would add important information that could influence the scientific and clinical communities.

The spread of SARS-CoV-2 has resulted in a global COVID-19 pandemic, and diagnostic practices for detecting this infection continue to be developed. Currently, SARS-CoV-2 is commonly detected by RT-PCR assays; however, limited reagent availability and the need for advanced laboratory facilities with restrictive biosafety levels can increase the technical complications associated with this procedure. In addition, the inconsistent recovery of respiratory samples has reduced the efficiency of timely disease diagnosis (Mathuria, Yadav, & Rajkumar, 2020).

Seroconversion is induced by the rapid change in antibody concentrations during the first two weeks of infection. 50% seropositivity can be detected at 11 days after infection and reaches 100% at 39 days post-infection (Zhao et al., 2020). Serological investigations can be used to recognize the occurrence of infection even when the virus is undetectable by RT–PCR, including the identification of asymptomatic infections (Long et al., 2020).

The quantitative detection of anti-S antibodies has been broadly utilized to describe the antibody response to COVID-19 in patients. A recent study investigated antibody responses in a group of COVID-19 patients and showed a strong relationship between the strength of the anti-S antibody response and patient survival (Sun et al., 2020).

The current study showed that COVID-19 patients harboured significant IgG antibody levels against the viral S protein in both severe and non-severe patients. These results agree with those of a previous study, which reported that IgG and IgM antibodies against the SARS-CoV-2 S protein were higher in severely ill patients than in non-severely ill patients (Sun et al., 2020). An association between higher antibody concentrations and increased disease severity was also reported by Jiang, who found that higher levels of the anti-Spike IgG antibody were associated with disease severity (Jiang et al., 2020).

Studies have shown that IgG antibodies against the SARS-CoV-2 S protein could be detected in the blood of more than 90% of all COVID-19 patients within 10–11 days after symptom onset (Amanat et al., 2020, Long et al., 2020b). How long these IgG antibodies remain detectable after recovery (Iyer et al., 2020) or whether they decline (Long et al., 2020) over time remains unclear.

The current study showed that all hospitalized severe patients had significant, cumulative anti-S IgG and IgM antibody levels compared with those levels in non-severe patients. Additionally, the results showed that 50% of severe males were positive for anti-S IgG, IgM, and IgA antibodies. In contrast, only 40% of severe female patients showed positivity for all three antibody isotypes. In the non-severe group, 43% of males were positive for all three isotypes, whereas 52% of females were positive for all the antibodies. Among the severe patients, only 6.5% were negative for all three antibody isotypes, whereas, among the non-severe patients, only one male (1%) was negative for all the antibodies. Among severe patients, only 17% were seropositive against IgG but were seronegative against IgM and IgA. In contrast, 26% of non-severe patients showed IgG seropositivity and were seronegative against IgM and IgA. Interestingly, 86% of male and 81% of female patients with severe COVID-19 were positive for both IgG and IgM anti-S antibodies but negative for IgA. In contrast, 32% of male and 28.5% of female non-severe patients were positive for both IgG and IgM but negative for IgA. Our results were consistent with those of another study conducted in Saudi Arabia, which showed a similar trend using neutralization antibody titres instead of antibody concentrations (Hashem et al., 2020).

The production of IgA antibody was not observed in the majority of subjects with seropositivity for the IgG antibody, which was expected because IgA tends to be secreted during the early stages of the disease. The current results are in agreement with a study that showed that human IgA antibodies are repeatedly measurable earlier than the presence of SARS-CoV-2–specific IgG antibodies, suggesting a functional role for IgA antibodies in primary virus neutralization (Sterlin et al., 2021). Additionally, another study suggested that compared with IgG antibodies, the SARS-CoV-2-specific IgA antibody might play a significant independent role in the development of protective mucosal immunity (Ejemel et al., 2020).

These humoral immune variations reflect differences in the immune response among patients manifesting the same degree of disease severity, which may be due to genetic differences, as previously suggested (Cao et al., 2020). In addition, variations may be due to the timing of sample acquisition relative to the timing of infection, as the date of the symptom onset was not recorded. The current study showed a higher degree of seronegativity against the IgA antibody relative to the seropositivity against IgG and IgM. Our results are supported by those of a recent study that reported the first seroconversion day for IgA as 2 days following the onset of early symptoms, and the initial seroconversion days for IgM and IgG were both 5 days after symptom onset (Yu et al., 2020). Therefore, the large proportion of the patients in the current study that showed seronegativity against IgA suggested the potential decline in serum IgA concentrations during the disease time course.

A recent study showed that SARS-CoV-2 provokes a strong humoral immune response, including the production of virus-specific antibodies of the IgG, IgM, and IgA isotypes (Long et al., 2020). Patients were reported to achieve seroconversion and produce measurable antibody concentrations approximately 15 days after symptom onset, whereas the kinetics of IgG and IgM production were not consistently associated with disease onset (Liu et al., 2021). A newly described investigation of an intranasal vaccination approach using a Middle East respiratory syndrome (MERS)-derived vaccine demonstrated the advantageous characteristics of the IgA antibody (Kim, Kim, & Chang, 2019). The mechanism underlying the virus-specific IgA response to SARS-CoV-2 infections in humans remains poorly studied (Sterlin et al., 2021).

The current results showed a strong positive correlation between IgG and IgM antibody levels and between IgM and IgA antibody levels. Therefore, IgG antibody concentrations were significantly higher than those for the other two isotypes. The specific anti-S IgG antibody commonly persists for a longer time and reflects the humoral immune memory against the virus, which is crucial for protection against reinfection (Mahallawi, 2020). Interestingly, the present results showed that among both severe and non-severe male patients, IgG antibody levels were higher than IgM levels, which were significantly higher than IgA levels, and a similar trend was observed in both severe and non-severe female patients. Moreover, a recent study showed that specific anti-S IgG antibody seropositivity continues in recovered COVID-19 patients up to a hundred days' post-infection(W. Mahallawi, Alzahrani, & Alahmadey, 2021).

Vaccine development is an urgent necessity to prevent COVID-19 and reduce the complications associated with the public spread of SARS-CoV-2 (Heaton, 2020).

A recent study showed that neither the quantity nor functionality of antibody responses were associated with disease severity and outcome in adults, although differences were observed between the paediatric cohorts (Pierce et al., 2020).

RT–PCR-based viral RNA detection is sensitive and can successfully verify early SARS-CoV-2 infection (Zou et al., 2020). The data has demonstrated that virus-specific antibody detection against COVID-19 can serve as an auxiliary method for the analysis of suspected cases with negative RT–PCR results. In a study conducted on blood donors, none of whom were aware of previous virus infection, more than 19% of all donors presented positive anti-SARS-CoV-2 IgG antibodies (Mahallawi & Al-Zalabani, 2020). This result highlights the significance of using serological testing to obtain more careful estimates of the magnitude of the COVID19 pandemic.

The current study has several limitations. First, the samples were not tested for virus neutralization; therefore, the neutralizing activities of the detected IgG antibodies were not verified. Second, due to the small sample size of patients in severe condition, the relationship between antibody responses and the clinical disease course was difficult to determine. Third, no sequential patients’ samples were available to investigate the kinetics of the humoral immune responses relative to the clinical and virological features of each patient. Finally, no specific date of symptom onset was recorded in the hospital records.

Further studies of the immune responses should be performed, investigating other cellular immunity markers with samples collected at different time points, which may provide insights into how the host immune response contributes to diverse clinical outcomes.

## Declaration of Competing Interest

The authors declare that they have no known competing financial interests or personal relationships that could have appeared to influence the work reported in this paper.
